# Long Non-Coding RNAs and p53 Regulation

**DOI:** 10.3390/ijms131216708

**Published:** 2012-12-06

**Authors:** Antonella Baldassarre, Andrea Masotti

**Affiliations:** Gene Expression-Microarrays Laboratory, IRCCS-Bambino Gesù Children’s Hospital, Rome 00165, Italy; E-Mail: antonella.baldassarre@opbg.net

**Keywords:** long non-coding RNAs, p53 pathway, LincRNA-p21, PANDA, H19, MEG3, LincRNA-EPS

## Abstract

The advent of novel and high-throughput sequencing (next generation) technologies allowed for the sequencing of the genome at an unprecedented depth. The majority of transcribed RNAs have been classified as non-coding RNAs. Among them, long non-coding RNAs (lncRNAs) are emerging as important regulators in many biological processes. Here, we discuss the role of those lncRNAs which are under the control of p53 or that are able to regulate its activity, due to the central role of p53 pathway in many conditions. We also briefly discussed the emerging need of having novel strategies and computational tools to completely unravel the multifaceted roles of lncRNAs and to pave the way to the development of novel diagnostic and therapeutic applications based on these peculiar molecules.

## 1. Introduction

In biology, the classical view of gene expression and regulation has been focused around protein-coding genes via the central dogma of DNA → mRNA → protein. However, over the past decade, evidence from numerous next generation sequencing (NGS) experiments suggested that the evolution of developmental processes, which regulate the complexity of the organism, is mainly due to the expansion of the regulatory potential of the non-coding portions of the genome [1]. In complex organisms, the portion of the genome responsible for coding proteins constitutes approximately 1.5%, while many regulatory elements are transcribed into non-coding RNAs (ncRNAs). Indeed, the recent technological breakthrough and knowledge coming from these novel NGS techniques, established the importance of ncRNAs in the regulation of multiple major biological processes related to development, differentiation, and metabolism [2–4] and have forced us to radically reinterpret our understanding of the genome [5–7]. In contrast to the small ncRNAs, such as short interfering RNAs (siRNAs), microRNAs (miRNAs), and Piwi-interacting RNAs (piRNAs) which are highly conserved and involved in transcriptional and post-transcriptional gene expression regulation through specific base pairing with their targets, long non-coding RNAs (lncRNAs)—defined as transcribed RNA molecules greater than 200 nt in length—are poorly conserved and regulate gene expression by diverse mechanisms that are not yet fully understood [2,4,8–11].

In eukaryotes, lncRNAs have been implicated in many biological processes with different functional roles such as X chromosome inactivation [12,13], genomic imprinting [14], sub-cellular structural organization [15,16], telomere [17] and centromere organization [18,19], and nuclear trafficking [20]. Nowadays, it is well acknowledged that mammalian genomes encode numerous long non-coding RNAs [5,21–23] and that many of them are linked to fundamental biological processes and signaling network (*i.e.*, the p53 pathway). Huarte *et al*. have demonstrated that numerous lncRNAs are key constituents in the p53-dependent transcriptional pathway [24]. Moreover, they observed that some of these lncRNAs are bound by p53 in their promoter regions and sufficient to drive p53-dependent reporter activity that requires the consensus p53-binding motif, suggesting that these lncRNAs are *bona fide* p53 transcriptional targets.

In this review, we focused the interest on those lncRNAs related to p53 regulation (induced or activating), owing to the central role of p53 in many processes. We therefore considered and discussed LincRNA-p21, PANDA, H19, MEG3 lncRNA and LincRNA-EPS.

## 2. Long Non-Coding RNAs at the CDKN1A (p21) Locus

### 2.1. LincRNA-p21

**Figure 1:**
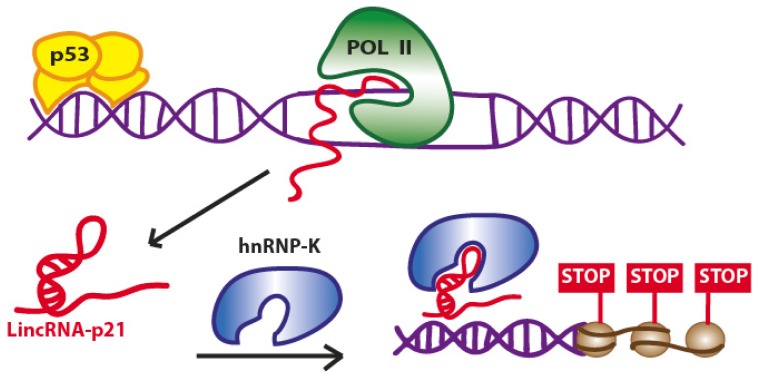
Proposed model for the role of LincRNA-p21 in the p53 transcriptional response. The induction of p53 activates the transcription of LincRNA-p21 by binding to its promoter (upper left). LincRNA-p21 binds to hnRNP-K and acts to repress genes that are downregulated as part of the canonical p53 transcriptional response. (Adapted from [24]).

The main study dealing with LincRNA-p21 and its role in p53 pathway has been reported quite recently [24]. The authors explored the functional roles of this lncRNA intrigued by its properties: genomic location ~15 kb upstream of the gene encoding the critical cell cycle regulator CDKN1A (p21), the presence of a consensus p53 motif for p53-dependent activation and conserved p53-dependent activation of this gene in both human and mouse cell models. Their studies emphasized a role for LincRNA-p21 in a p53-dependent apoptotic response after DNA damage. The authors further observed that siRNA-mediated inhibition of LincRNA-p21 affects the expression of hundreds of target genes, normally repressed by p53, in both the mouse embryonic fibroblasts (MEF) and in lung tumor cell line derived from mice expressing an oncogenic K-Ras mutation (KRAS). Strikingly, upon inhibition of either p53 or LincRNA-p21, the vast majority of these target genes were upregulated suggesting that this lncRNA acts as a downstream repressor in the p53 transcriptional response. Huarte *et al.* gained also mechanistic clues into how LincRNA-p21 functions to repress such a large subset of genes in the p53 transcriptional response. Biochemical experiments such as RNA-pulldown, native RIP, cross-linked RIP, and deletion-mapping experiments, allowed the authors to identify a specific interaction between the LincRNA-p21 and the heterogeneous nuclear ribonucleoprotein K (hnRNP-K) (Figure 1).

Moreover, they identified a highly conserved 780 nt 5′ region in the sequence of LincRNA-p21 which is required for hnRNP-K binding and subsequent induction of apoptosis. In fact, the two major phenotypic consequences of p53 pathway activation are the growth arrest and the induction of apoptosis [25]. In their work, the authors demonstrated the deregulation of many apoptosis and cell cycle regulator genes by p53 and LincRNA-p21. To mediate gene repression, a physical interaction between LincRNA-p21 and hnRNP-K is required, as the loss of hnRNP-K function induce a reactivation of the same genes repressed by p53 and LincRNA-p21. However, the precise mechanism by which LincRNA-p21 contributes to repression at specific loci has yet to be clarified.

### 2.2. PANDA (P21 Associated ncRNA DNA Damage Activated)

The long non-coding RNA PANDA (a ~1.5-kb transcript) is located ~5 kb upstream of the CDKN1A (p21) transcription start site, is evolutionarily conserved, specifically induced by DNA damage in a p53-dependent manner and mediates anti-apoptotic functions [26]. However, the expression of PANDA is not dependent on p21. It has been observed that PANDA depletion induced several genes encoding canonical activators of apoptosis, such as APAF1, BIK, FAS and LRDD [26].

**Figure 2:**
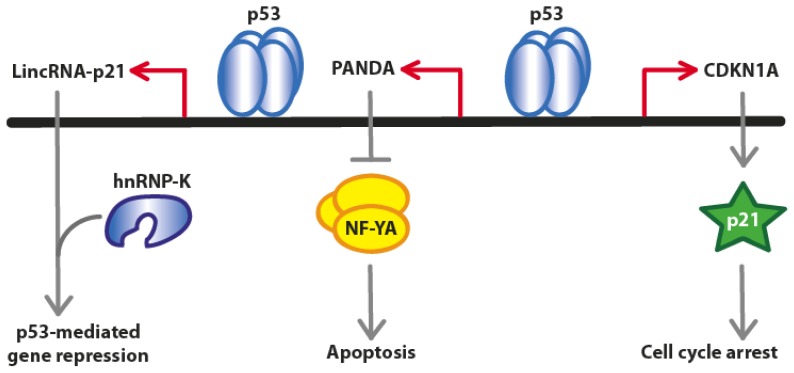
Model of coding and non-coding transcripts at the *CDKN1A* locus coordinating the DNA damage response. After DNA damage, p53 binding at the *CDKN1A* locus coordinately activates transcription of *CDKN1A* as well as non-coding transcripts PANDA and LincRNA-p21. CDKN1A mediates cell cycle arrest, PANDA blocks apoptosis through NF-YA, and LincRNA-p21 mediates gene silencing through recruitment of hnRPK. (Adapted from [26]).

Promoter regions of p53-dependent cell death genes are characterized by the presence of a binding site for the transcription factor NF-YA, which interacts with PANDA in a highly specific manner [27]. Moreover, the depletion of PANDA substantially increases the occupancy of NF-YA at pro-apoptotic target genes such as CCNB1, FAS, BBC3 (PUMA) and PMAIP1 (NOXA). Therefore, whereas CDKN1A mediates cell cycle arrest, PANDA promotes cell survival by interfering with the apoptotic gene expression program (Figure 2).

## 3. LincRNA-EPS

**Figure 3:**
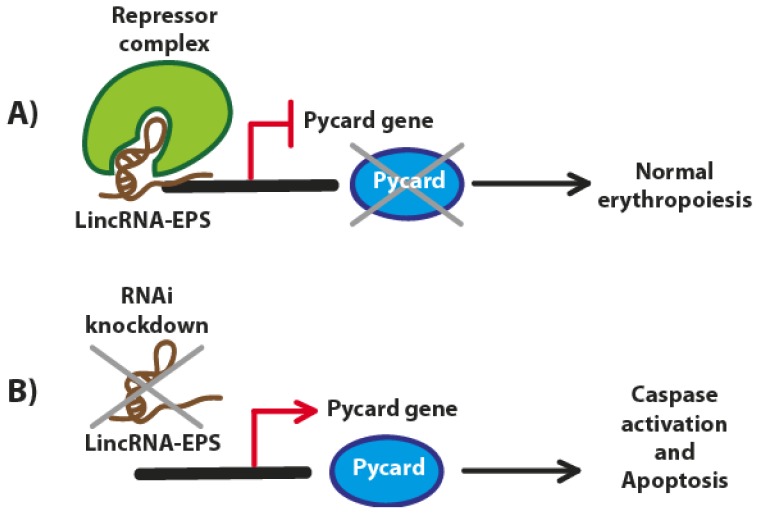
The potential role of LincRNA-EPS in erythropoiesis. (**A**) LincRNA-EPS represses Pycard gene to inhibit apoptosis of red blood cells progenitors. LincRNA-EPS may bind the Pycard gene and inhibit its transcription by recruiting transcriptional repressor complexes; (**B**) Knockdown of LincRNA-EPS allows the transcription of Pycard, which in turn activates intercellular caspases and induces apoptosis. (Adapted from [31]).

During erythropoiesis more than 400 putative lncRNAs are differentially expressed. One of these lncRNAs, named long intergenic non-coding RNA erythroid prosurvival (LincRNA-EPS), is induced during the terminal differentiation of murine erythroid cells and has been recently investigated [28]. LincRNA-EPS is a 2531-nt lncRNA originated from a DNA portion consisting of four exons and three introns, bearing a 5′ end cap structure and a 3′ poly(A) tail. LincRNA-EPS is strongly induced when erythroid precursors begin to synthesize hemoglobin and other lineage-specific proteins. LincRNA-EPS is expressed marginally in other hematopoietic lineages, indicating erythroid specificity. It has been observed that by knocking down LincRNA-EPS, an inhibition of differentiation and the promotion of apoptosis take place. Conversely, ectopic expression of this lncRNA can prevent apoptosis in mouse erythroid cells. Although we still do not know if this LincRNA-EPS is under the direct control of p53, LincRNA-EPS can repress the expression of the proapoptotic gene Pycard, inhibiting programmed cell death [28]. On the other hand, the induction of Pycard (also known as ASC, CARD5, TMS or TMS-1) has been alternatively reported by the same authors to be either dependent on p53 (after exposure to a genotoxic stress in the intrinsic mitochondrial pathway of apoptosis through a p53-Bax network) [29] or p53-independent (under hypoxic conditions in pancreatic cancer cells) (Figure 3) [30]. However, from a preliminary bioinformatics search we found that various p53 regulatory transcription factor binding sites are present in the Pycard gene promoter (data not shown).

To study the functional link between LincRNA-EPS and Pycard and how this lncRNA can inhibit apoptosis, Hu *et al.* employed microarrays technology to assess the gene expression of lineage-negative (Lin-) fetal liver cells, highly enriched for erythroid progenitors [32] and ectopically expressing LincRNA-EPS. The authors found that many genes involved in apoptosis were downregulated, and the proapoptotic Pycard gene was the most downregulated. These data suggested that Pycard can be one of the targets of LincRNA-EPS and its expression during normal erythropoiesis is inversely correlated with that of the LincRNA-EPS. Moreover, the overexpression of Pycard gene is able to inhibit the proliferation of erythroid cells to promote apoptosis and interfere with their terminal differentiation and enucleation.

The nuclear localization of LincRNA-EPS suggested that the gene expression regulation might occur through nuclear events such as epigenetic modifications, transcription, or mRNA splicing, or simply by its association with chromatin modifiers which repress the transcription of Pycard and the other genes promoting cell apoptosis. Finally, further studies aimed at discovering this lncRNA and other potentially associated factors in humans and novel and more powerful computational methods and prediction algorithms will surely help to identify all the actors involved in this mechanism.

## 4. p53 Regulation by lncRNAs

### 4.1. H19

The lncRNA H19 has been recently investigated and found upregulated in many tumors [33,34]. Moreover, it has been found that ectopic expression of H19 increased cell proliferation, whereas H19 silencing (siRNAs) contributed to apoptosis in human gastric cancer cells (AGS cells) [35]. In the latter study, the authors aimed also to investigate if the molecular mechanism by which H19 increases gastric cancer cell growth required p53 activation.

In fact, several groups have studied the link between H19 and p53 [36,37]. Dugimont *et al.* demonstrated that the H19 promoter is efficiently repressed by p53 [37]. By immunoprecipitation and RNA pulldown experiments, Yang *et al.* demonstrated the association of H19 and p53, and by transfecting H19 in AGS cells they observed a significant decrease of p53 activity, suppressing also the protein level of the p53 target Bax. Therefore, these data emphasize the fact that the upregulation of the lncRNA H19 contributes to tumorigenesis through p53 activity regulation, at least in gastric cancers.

### 4.2. MEG3 lncRNA

**Figure 4:**
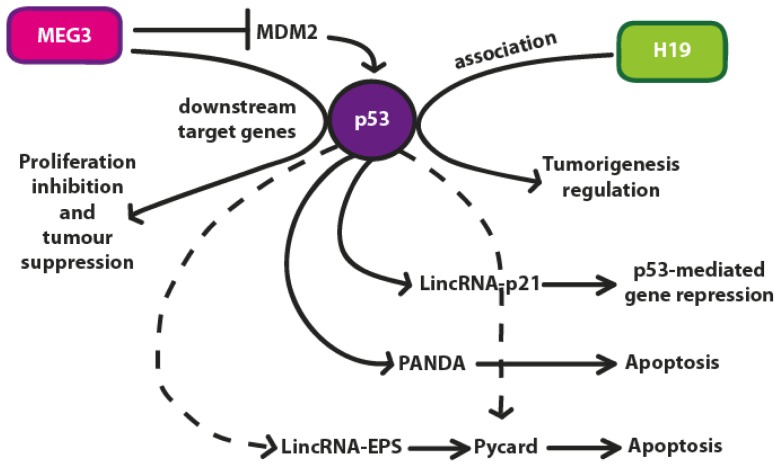
In the p53 pathway, MEG3 may activate p53 directly by RNA-protein interaction or indirectly by suppressing MDM2, resulting in selective activation of p53 downstream targets such as GDF-15 with both anti-proliferative and tumor suppressive functions. The association of H19 with p53 determines tumorigenesis regulation. The role of the lncRNAs LincRNA-p21, PANDA and LincRNA-EPS have been discussed in the text.

The transcript of the maternally expressed gene 3 (MEG3) is reported to have several isoforms originating from the human MEG3 gene which contains 10 exons [38]. The length of the different isoforms is variable although their mean value is ~1700 nt. MEG3 lncRNA is expressed in many normal tissues (*i.e.*, human pituitary, including normal gonadotroph cells) but absent in a continuously increasing list of primary human tumors and tumor cell lines [39,40]. MEG3 is expressed during development with higher levels in the paraxial mesoderm, the developing central nervous system, and the epithelia of salivary glands, pancreas and kidney. It is also expressed in adult mouse adrenal and pituitary glands and brain [41]. Gene deletion, promoter hypermethylation and hypermethylation of other intergenic regions are the most probably causes for MEG3 lncRNA loss in these cases. MEG3 lncRNA induces an accumulation of p53 protein, stimulating the transcription from a p53-dependent promoter and selectively regulates the expression of p53 target genes (Figure 4) [42]. In conclusion, MEG3 lncRNA can be considered a novel tumor suppressor lncRNA [43].

Interestingly, Zhang *et al.* pointed out for the first time that the folding structure of MEG3 lncRNA is more important to its biological function than its primary sequence [38]. In fact, by examining the secondary folding structures of the main MEG3 lncRNA domains, which they identified and called M1, M2 and M3, Zhang *et al.* assessed that p53-mediated transactivation varies between these different isoforms and that M2 domain has a critical role. As a direct consequence of their results, the most fascinating observation is that also the folding structures of lncRNAs are important factors to consider as they are intimately linked to their functions. We can imagine that, like proteins, also lncRNAs can fold into tertiary structures determining different outcomes and functions. Further crystallographic and RNA-protein studies will surely shed light on these interesting, but still unanswered, questions.

## 5. Conclusions

Many studies have recently identified and discussed the role of numerous lncRNAs which are involved in p53 regulation or have a role in carcinogenesis or cancer growth [40,44–48]. These lncRNAs are emerging as important regulators of gene expression by acting synergistically with other factors in many different and crucial mechanisms. The interplays between lncRNAs and p53 regulation has been discussed in this review and summarized in Figure 4.

The advent of next-generation sequencing techniques has paved the way to important discoveries also in the field of non-coding RNAs, but the exact biological role of this regulatory molecules has still to be clarified. Notwithstanding, many studies are beginning to shed light on lncRNAs and their mechanisms and more information are expected to arise in the next few years.

In this brief review, we emphasized only the role of a selection of these lncRNAs, in particular of those molecules involved in p53 pathway and in its regulation owing to the central importance of this pathway in many conditions. We are aware that many other lncRNAs may play a role in this pathway, although this role is not completely unraveled so far. Moreover, little is known about the expression level (*i.e.*, the threshold) required to a lncRNA to function properly, or the relative abundance in various tissues. To partially answer these questions, a novel integrative approach consisting in a computational approach integrating RNA-seq data with available annotation resources has been recently reported [49]. The result is a reference catalog (Human Body Map lincRNAs) [50] of 8195 human lincRNAs with information about their expression levels in different tissues and linked to the expression of their neighboring genes.

Finally, the bioinformatics tools that will be developed in the near future will surely help researchers to fill the knowledge gaps and to face the present challenges [51]. We believe that the world of lncRNAs, as it was for the “junk” non-coding RNAs such as miRNAs, will open unexpected possibilities, not only for biological and biomedical research, but also for translational research and innovative clinical “theranostics” (therapeutics and diagnostics) applications.
